# Surgery for Unresectable Stage IIIC and IV Melanoma in the Era of New Systemic Therapy

**DOI:** 10.3390/cancers12051176

**Published:** 2020-05-07

**Authors:** Stephanie A. Blankenstein, Maureen J. B. Aarts, Franchette W. P. J. van den Berkmortel, Marye J. Boers-Sonderen, Alfons J. M. van den Eertwegh, Margreet G. Franken, Jan Willem B. de Groot, John B. A. G. Haanen, Geke A. P. Hospers, Ellen Kapiteijn, Djura Piersma, Rozemarijn S. van Rijn, Karijn P. M. Suijkerbuijk, Albert J. ten Tije, Astrid A. M. van der Veldt, Gerard Vreugdenhil, Michel W. J. M. Wouters, Alexander C. J. van Akkooi

**Affiliations:** 1 Department of Surgical Oncology, Netherlands Cancer Institute-Antoni van Leeuwenhoek, Plesmanlaan 121, 1066 CX Amsterdam, The Netherlands; s.blankenstein@nki.nl (S.A.B.); m.wouters@nki.nl (M.W.J.M.W.); 2 Department of Medical Oncology, Maastricht University Medical Centre, P. Debyelaan 25, 6229 HX Maastricht, The Netherlands; mjb.essers.aarts@mumc.nl; 3 Department of Internal Medicine, Zuyderland Medical Centre Geleen-Heerlen, Dr. H. van der Hoffplein 1, 6162 BG Sittard-Geleen, The Netherlands; f.vandenberkmortel@zuyderland.nl; 4 Department of Medical Oncology, Radboud University Medical Centre, Geert Grooteplein Zuid 10, 6525 GA Nijmegen, The Netherlands; Marye.Boers-Sonderen@radboudumc.nl; 5 Department of Medical Oncology, Cancer Center Amsterdam, Amsterdam UMC, location VU University Medical Center (VUmc), Cancer Center Amsterdam, De Boelelaan 1118, 1081 HZ Amsterdam, The Netherlands; vandeneertwegh@vumc.nl; 6 Institute for Medical Technology Assessment, Erasmus University, Burgemeester Oudlaan 50, 3062 PA Rotterdam, The Netherlands; franken@imta.eur.nl; 7 Oncology Center Isala, Isala, Dokter van Heesweg 2, 8025 AB Zwolle, The Netherlands; j.w.b.de.groot@isala.nl; 8 Department of Medical Oncology, Netherlands Cancer Institute-Antoni van Leeuwenhoek, Plesmanlaan 121, 1066 CX Amsterdam, The Netherlands; j.haanen@nki.nl; 9 Department of Medical Oncology, University Medical Centre Groningen, University of Groningen, Hanzeplein 1, 9713 GZ Groningen, The Netherlands; g.a.p.hospers@umcg.nl; 10 Department of Medical Oncology, Leiden University Medical Centre, Albinusdreef 2, 2333 ZA Leiden, The Netherlands; h.w.kapiteijn@lumc.nl; 11 Department of Internal Medicine, Medisch Spectrum Twente, Koningsplein 1, 7512 KZ Enschede, The Netherlands; D.Piersma@mst.nl; 12 Department of Internal Medicine, Medical Center Leeuwarden, Henri Dunantweg 2, 8934 AD Leeuwarden, The Netherlands; Rozemarijn.van.Rijn@ZNB.NL; 13 Department of Medical Oncology, University Medical Center Utrecht, Heidelberglaan 100, 3584 CX Utrecht, The Netherlands; K.Suijkerbuijk@umcutrecht.nl; 14 Department of Internal Medicine, Amphia Hospital, Molengracht 21, 4818 CK Breda, The Netherlands; AtenTije@amphia.nl; 15 Department of Medical Oncology, Erasmus Medical Center, Doctor Molewaterplein 40, 3015 GD Rotterdam, The Netherlands; a.vanderveldt@erasmusmc.nl; 16 Department of Internal Medicine, Maxima Medical Center, De Run 4600, 5504 DB Veldhoven, The Netherlands; g.vreugdenhil@mmc.nl; 17 Dutch Institute for Clinical Auditing, Rijnsburgerweg 10, 2333 AA Leiden, The Netherlands

**Keywords:** metastatic melanoma, surgery, systemic therapy, Dutch Melanoma Treatment Registry

## Abstract

Opportunities for surgical treatment in metastatic melanoma patients have re-emerged due to the development of novel systemic therapeutics over the past decade. The aim of this study is to present data on outcomes of surgery in patients with unresectable stage IIIC and IV melanoma, who have previously been treated with immunotherapy or targeted therapy. Data was extracted from the Dutch Melanoma Treatment Registry (DMTR) on 154 patients obtaining disease control to systemic therapy and undergoing subsequent surgery. Disease control was defined as a complete response (CR), which was seen in 3.2% of patients; a partial response (PR), seen in 46.1% of patients; or stable disease (SD), seen in 44.2% of patients. At a median follow-up of 10.0 months (interquartile range 4–22) after surgery, the median overall survival (OS) had not been reached in our cohort and median progression-free survival (PFS) was 9.0 months (95% CI 6.3–11.7). A CR or PR at first follow-up after surgery was associated with both a better OS and PFS compared to stable or progressive disease (*p* < 0.001). We conclude that selected patients can benefit from surgery after achieving disease control with systemic therapy.

## 1. Introduction

Historically the prognosis of patients with unresectable stage III and IV melanoma has been poor, with a median overall survival (OS) of only 6.2 months [1,2]. Some patients with oligometastatic melanoma (up to three lesions) can be treated by surgery and achieve long-term survival of around 40% at 5 years, especially those with only one lesion and a long interval between their melanoma and the development of stage IV disease [3,4]. However, it is difficult to select the patients that would benefit from such surgery.

Over the past decade the development of new systemic options has drastically changed the treatment of metastatic melanoma and therefore the prognosis of these patients. Immune checkpoint inhibitors (ICI) targeting PD-1 and CTLA-4, either as monotherapy or combined treatment, have shown response rates of approximately 40% to 60% respectively, improving progression-free and overall survival [5,6,7,8,9,10,11,12,13,14,15,16,17,18]. BRAF and MEK inhibitors in patients with BRAF-mutated melanoma have even higher overall response rates (70%), but fewer durable responses due to the development of resistance [19,20,21,22,23,24,25]. This evolution in therapeutic options has also presented new opportunities for surgery in this group of patients.

In some patients experiencing a durable partial response to systemic therapy, resection of the remaining lesion(s) can contribute to obtaining a complete response. Additionally, in patients with a partial or complete response developing oligoprogression, resection of the progressive lesion(s) may be performed. However, although these surgeries are already being performed in clinical practice, little evidence has been presented to support this treatment approach.

The aim of this population-based study is to present data on the incidence and outcomes of surgery in patients with unresectable stage III and IV melanoma, who have been treated with immunotherapy or targeted therapy (TT) prior to surgery (no first line surgery included), to provide an insight into which patients may benefit from surgery after obtaining disease control with systemic therapy.

## 2. Patients and Methods

Data were retrieved from the Dutch Melanoma Treatment Registry (DMTR). In this nationwide prospective database, all Dutch patients undergoing treatment for unresectable stage IIIC and IV melanoma are included. This registry was set up to monitor the safety and the outcomes of the novel treatments [26]. Registration in the DMTR is a prerequisite for reimbursement, assuring nationwide coverage. In compliance with Dutch regulations, the DMTR was approved by the medical ethical committee and was not subject to the Medical Research Involving Human Subjects Act. Patients were offered an opt-out option. In the current study we included patients from the database who had commenced treatment between the start of the registry (July 2012) and July 2017 to assure sufficient follow-up at data extraction in April 2018.

### 2.1. Patients

Patients who had surgery after obtaining disease control with systemic therapy were selected from the registry. Disease control was defined as stable disease (SD), partial response (PR) or complete response (CR) as the best response to systemic therapy. These responses were non-confirmed investigator-assessed responses, retracted from follow-up data registered in the database, therefore this cannot be considered the same as RECIST-measured responses. Progressive disease (PD) was allowed as a most recent status of disease prior to surgery if these patients initially had a SD, PR or CR as their best response. Patients who had primarily progressive disease and underwent surgery were excluded, as we considered that this would include a substantial number of palliative surgeries for symptomatic patients, which was not the focus of this study.

Patients with uveal and mucosal melanoma were excluded, since these subtypes differ in biologic behavior and in their responses to immunotherapy and targeted therapy. Moreover, patients presenting with brain metastases were excluded from this study, since these patients generally have a different prognosis.

### 2.2. Statistical Analysis

Data were analyzed using IBM SPSS Statistics, version 25 (IBM Corp., Armonk, NY, USA). Descriptive statistics were used to assess patient, tumor, systemic therapy, surgery and follow-up characteristics. The baseline for patient and tumor characteristics was set at start of systemic therapy. Characteristics of patients treated with ICI were compared to patients treated with targeted therapy using the chi-square test (categorical variables) and *t*-Test (continuous variables). Kaplan-Meier methods and log-rank tests were applied to calculate and compare progression-free survival (PFS) and OS and Cox regression models were used to analyze the influence of different variables. Variables that were (borderline) significant in the univariate analyses (and consisted of sufficient patient numbers) were used in the multivariate Cox regression models. PFS and OS were defined as the time between surgery and first disease progression or death, respectively. Patients not experiencing an event were censored at the time of last follow-up.

## 3. Results

### 3.1. Patient and Tumor Characteristics

At the time of data extraction, the DMTR database consisted of 3959 patients, of whom 876 had undergone surgery during their treatment and 463 patients received systemic treatment prior to surgery. After selecting patients obtaining disease control (SD/PR/CR) with systemic therapy, 154 patients remained. The baseline characteristics of these 154 patients and the treatment they received are listed in Table 1. The median age of our study population was 58, ranging from 24 to 87. The vast majority of patients had a good performance score, WHO zero or one (91.6%), and a normal lactate dehydrogenase (LDH) level (74.7%), which is a known prognostic factor for survival in metastatic melanoma patients [27]. The percentage of patients with a BRAF mutation was slightly higher than usual in the general patient population (68.8%), most likely due to the selection for response to systemic treatment. Most patients presented with distant metastases: 44.2% with distant metastases only and 34.4% with both distant and locoregional metastases compared to 21.4% of patients presenting with only locoregional metastases. A substantial proportion of patients presenting with distant metastases had nodal or subcutaneous metastases: 58.2% and 37.2%, respectively. The number of metastases before the start of systemic treatment was poorly documented, with missing data in 37% of patients, but most patients presented with multiple (>10) lesions.

No differences were seen in baseline characteristics between patients treated with immunotherapy and targeted therapy, except for the location of the metastases (Table 1). More patients treated with targeted therapy had locoregional metastases only, compared to patients treated with ICI (31.1% vs. 11.4%, *p* = 0.007), of whom a larger percentage were treated for distant metastases (53.2% vs. 32.8%).

### 3.2. Treatment

Surgery was performed after the first line of systemic treatment in 69.5% of patients. Little over half of patients (51.3%) were treated with ICI, 39.6% with targeted therapy, and 9.1% of patients with other treatment (in trials) or the given treatment was unknown. Of patients with a BRAF mutation, the majority received targeted therapy (57.5%), the remainder received either immunotherapy (34.0%) or other treatments (8.5%). Patients receiving immunotherapy were roughly evenly divided between anti-PD1 directed therapy (48.1%) and anti-CTLA4 therapy (44.3%) and only a small percentage (7.6%) were treated with combination ICI. Of patients receiving targeted therapy, about half (50.8%) were treated with a BRAF inhibitor alone and in the remaining patients (49.2%) it was combined with a MEK inhibitor.

### 3.3. Response

#### 3.3.1. Best Response

Only a very small proportion of patients (3.2%) achieved a complete response as the best response to systemic treatment prior to surgery and the fractions of patients obtaining a partial response and stable disease as a best response were similar (46.1% and 44.2%).

#### 3.3.2. Most Recent Disease Status Prior to Surgery

The most recently reported status of disease prior to surgery was PD in 46.1% of patients, versus 29.2% of patients with SD and 18.8% with a PR before surgery. As shown above, the best response to systemic therapy was not necessarily the same as the most recent status of disease prior to surgery. For example, if a patient had a CR upon systemic therapy, but developed a recurrence and was operated for this lesion in due course, then the best response was CR, but the most recent status of disease prior to surgery was classified as PD.

In the vast majority of patients, subcutaneous (39.6%) or lymph node (42.9%) metastases were resected and few serious complications occurred.

#### 3.3.3. First Evaluation after Surgery

In total, 31.8% of patients achieved a complete response at the first new evaluation after surgery, but 16.9% of patients had progressive disease at first follow-up after surgery. A summary of all responses is shown in Figure S1 and Table S1.

### 3.4. Survival Outcomes

**Figure 1:**
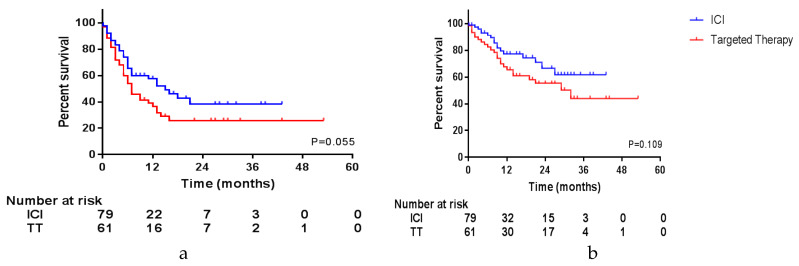
Survival per type of systemic therapy. (**a**) Progression-free survival; (**b**) overall survival.

At a median follow-up of 10.0 months (interquartile range 4–22) after surgery, the median OS had not been reached in our cohort (1-year OS was 70% and 2-year OS 59%) and median PFS was 9.0 months (95% CI 6.3–11.7). Figure 1a,b show Kaplan–Meier curves of the PFS and OS of the patients treated with ICI and targeted therapy separately. Figure S2 shows the PFS and OS of the entire cohort. The time to next treatment has not been shown, since this was similar to the PFS.

**Figure 2:**
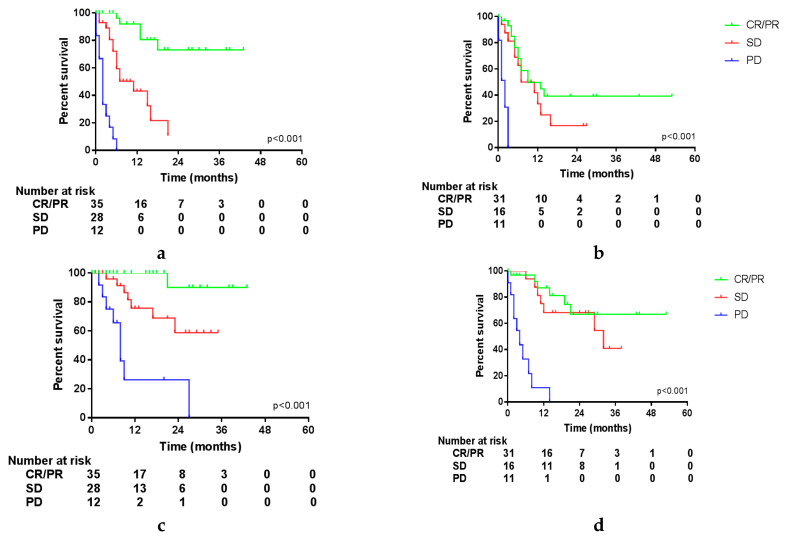
Survival per status of disease at first follow-up after surgery. (**a**) Progression-free survival (PFS) in patients treated with ICI; (**b**) PFS in patients treated with targeted therapy; (**c**) overall survival (OS) in patients treated with ICI; (**d**) OS in patients treated with targeted therapy.

Since survival could be influenced by the response to systemic treatment, we compared Kaplan–Meier curves of these different variables. The influence of these variables was tested in the entire cohort and in patients treated with either ICI or targeted therapy separately. OS and PFS of the entire cohort were not influenced by the best response to systemic treatment. However, in patients treated with ICI, a trend was seen in PFS, favoring patients with a PR compared to patients with SD (Figure S3; CR was not shown due to the very limited number of patients). The most recent status of disease prior to surgery had an impact on PFS and OS. Patients with PD before surgery had a median PFS after surgery of 5.0 months and a median OS of 17 months, compared to a not-reached median PFS and OS in patients with a PR (*p* = 0.009 and *p* = 0.004). As shown in Figure S4, this impact is seen in patients treated with targeted therapy and is even more pronounced in patients treated with ICI. Moreover, the status of disease determined at first evaluation after surgery had a significant impact on OS and PFS in the entire cohort and both treatment groups. This is shown in Figure 2 and Figure S5, with a median OS of 7 months in patients with PD compared to 29 months in patients with SD and not reached in patients with a CR or PR after surgery (*p* < 0.001 in both groups). Unfortunately, further follow-up data were missing in a substantial portion of the patients achieving a CR after surgery, and since the outcomes did not significantly differ from patients achieving a PR (*p* = 0.966), these groups were combined.

**Figure 3:**
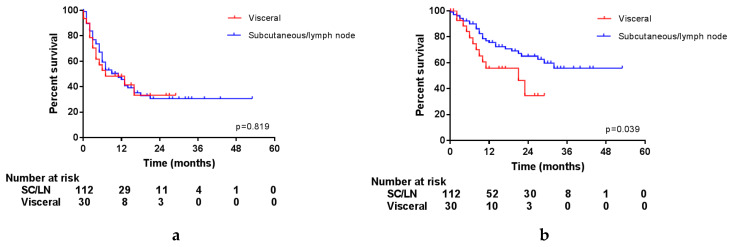
Survival per location of surgery. (**a**) Progression-free survival; (**b**) overall survival.

Interestingly, the location of the resected lesions had an impact on OS, but not on PFS, as is displayed in Figure 3. Unfortunately, it was not possible to differentiate between distant or locoregional subcutaneous and lymph node metastases in this database. However, this shows that after resection of a lymph node or subcutaneous metastasis (either locoregional or distant), patients do experience progression as quickly as after resection of a visceral metastasis, but this does not seem to influence the OS of these patients.

Other factors that could influence PFS and OS were compared in a univariate Cox regression analysis. Table 2a shows factors that had a significant impact on PFS, OS or both and Table S2 shows all factors. In the univariate analysis type of systemic therapy had no statistically significant impact on PFS or OS, however, a trend favoring ICI might be visible, so this factor was included in further analyses. 

All factors that showed (borderline) significance in both PFS and OS univariate analyses were used to perform a multivariate Cox regression analysis. These results are shown in Table 2b. Disease status after surgery is the most convincing factor, which has a significant impact on both PFS and OS. Moreover, immunotherapy prior to surgery was associated with a PFS and OS benefit compared to targeted therapy, when corrected for other factors in the multivariate analysis.

## 4. Discussion

We found that in metastatic melanoma patients obtaining disease control with systemic therapy and undergoing subsequent surgery, the most convincing factor associated with a more favorable outcome was the disease status (CR or PR) at first follow-up after surgery. Moreover, immunotherapy compared to targeted therapy and a duration of systemic therapy of over 3 months seemed to have a positive effect on prognosis.

OS and PFS in our cohort seem to be better than historically, with a median PFS after surgery of 9 months and OS not reached. Howard et al. retrospectively studied patients who had surgery with or without systemic therapy versus systemic therapy alone for stage IV melanoma, all of whom were initially treated in the MSLT-1 trial [4]. They described a survival benefit for surgery with or without systemic therapy versus systemic therapy alone (median OS of 15.8 vs. 6.9 months and 4-year survival of 20.8% vs. 7.0%). However, in this study by Howard, most patients had limited disease and in our cohort the majority of patients commenced systemic treatment with >10 lesions. Sosman et al. prospectively analyzed patients undergoing complete resection of stage IV melanoma and found a median recurrence-free survival of 5 months and median OS of 21 months [3]. The fact that both studies were conducted in an era without the current effective systemic therapy options explains the difference in outcomes with our cohort. Moreover, it must be noted that median follow-up in the Sosman study was substantially longer than in our cohort (5 years vs. 10 months). Follow-up in our cohort is limited because patients were treated with (novel) systemic therapy first and follow-up was measured from surgery and not from the start of systemic therapy.

The selection of patients who could benefit from surgery is crucial and, in this study, we found that expected residual tumor after surgery could be an important selection criterion. Bello et al. described a similar finding, as they had studied 237 patients who had surgery after immunotherapy. They found that a resection to no evidence of disease (NED) was associated with a better survival than residual disease after resection (OS not reached versus 10.8 months, 95% CI 7.3–14.8, *p* < 0.0001) [28]. Additionally, they described that OS was associated with the response to systemic therapy: patients with a response or oligoprogression did significantly better than patients with multiple progressive lesions. Unfortunately, in our database, it was not registered how many sites of progression were present in PD cases before surgery. Therefore, we could not distinguish oligoprogression from multiple progressive lesions. Klemen et al. studied patients resected to NED or non-progressive residual disease (NPRD) after progression following immunotherapy and showed a substantial 5-year disease-specific survival of 60% and no significant differences between survival in NED and NPRD patients [29]. They stratified patients for patterns of failure and patients with progression in established tumors had a significant better PFS than patients with new metastases (3-year PFS of 70% versus 6%, *p* = 0.001). Thus, other studies seem to confirm that the expected presence of a residual tumor after resection may be an important factor in selecting the correct patients for surgery.

Imaging may be helpful to select patients prior to surgery. Tan et al. described that complete metabolic response on FDG-PET could be useful in predicting long-term benefits and could guide the discontinuation of therapy in metastatic melanoma patients treated with immunotherapy [30]. Perhaps this could also be used in selecting patients for surgery. However, we are unable to test this hypothesis in our database, since these data were not collected.

This is one of the limitations of our study: the DMTR contains valuable information on metastatic melanoma patients, but data in this study is limited to the data that were provided by the registry. There is no possibility to find additional unregistered clinical data, nor to perform additional translational analyses. For example, no RECIST response measurements are registered and therefore information on treatment response has to be extracted from the status of disease at follow-up. Moreover, since this is a retrospective study using prospective collected data, selection bias may still occur. The strengths of using data extracted from the DMTR are its nationwide coverage and prospective data collection by trained data managers of real-world data.

The rapid developments in treatment options in recent years have caused some heterogeneity in the registered data. For example, the type of systemic treatment patients in our cohort received does not completely reflect current practice. Anti-CTLA-4 agent ipilimumab was the first immune checkpoint inhibitor available, but anti-PD1 has proven to be superior to ipilimumab and is currently the first choice for most patients [9,13,15]. However, in our cohort, 43.6% of patients were treated with ipilimumab. Moreover, the addition of a MEK inhibitor was shown to improve response rates over a BRAF inhibitor alone, so this has become standard of care [9,19,20,23,24]. During the initial years of the registry, MEK inhibitors were not yet reimbursed and therefore half of the patients in our cohort were treated with a BRAF inhibitor alone.

Targeted therapy is known for its high and quick response rates, where ICI is known to have more durable responses. This may cause a selection bias because, in patients with a worse baseline situation, targeted therapy may be preferentially chosen over ICI. However, when comparing baseline characteristics (LDH, performance status, etc.) between patients treated with targeted therapy versus ICI in our cohort, there are no significant differences. Thus, even after correcting for a potential selection bias, surgery after ICI seems to be superior to surgery after TT treatment.

Although several retrospective studies, including ours, do suggest a benefit for surgery after a response to immunotherapy and/or targeted therapy, further studies are warranted. If patients have a deep response with only limited residual lesion(s), stopping therapy, continuing therapy and/or resection can be possible approaches. A randomized trial would be needed to appropriately address this issue.

## 5. Conclusions

Disease status after surgery is the most important prognostic factor for OS and PFS for unresectable stage III/stage IV melanoma patients. Therefore, we recommend that in patients with multiple metastases, surgery is only considered after systemic therapy, when a partial or complete response can be achieved after the resection.

## Figures and Tables

**Table 1:** Patient, tumor and treatment characteristics.

Characteristic	Total (*n* = 154) *n* (%)	ICI (*n* = 79) *n* (%)	TT (*n* = 61) *n* (%)	*p* ^1^
Age, years				0.452
Median	58	62	59	
Range	24–87	24–80	34–87	
Sex				0.444
Female	74 (48.1)	35 (44.3)	31 (50.8)	
Male	80 (51.9)	44 (55.7)	30 (49.2)	
WHO performance status				0.543
0	101 (65.6)	52 (65.8)	38 (62.3)	
1	40 (26.0)	21 (26.6)	16 (26.2)	
2	4 (2.6)	3 (3.8)	1 (1.6)	
3	1 (0.6)	0 (0.0)	1 (1.6)	
Unknown	8 (5.2)	3 (3.8)	5 (8.2)	
Location primary				0.431
Extremity	61 (39.6)	32 (40.5)	23 (37.7)	
Trunk	45 (29.2)	20 (25.3)	20 (32.8)	
Head/Neck	10 (6.5)	4 (5.1)	6 (9.8)	
Acral	9 (5.8)	4 (5.1)	4 (6.6)	
MUP ^2^	27 (17.5)	17 (21.5)	8 (13.1)	
Unknown	2 (1.3)	2 (2.5)	0 (0.0)	
Type				0.637
Superficial spreading	63 (40.9)	27 (34.2)	28 (45.9)	
Nodular	27 (17.5)	13 (16.5)	10 (16.4)	
Acral lentiginous	8 (5.2)	5 (6.3)	3 (4.9)	
Lentigo maligna	1 (0.6)	0 (0.0)	1 (1.6)	
Desmoplastic	1 (0.6)	1 (1.3)	0 (0.0)	
Other	5 (3.2)	4 (5.1)	1 (1.6)	
Unknown	20 (13.0)	10 (12.7)	10 (16.4)	
Missing (MUP ^2^)	29 (18.8)	19 (24.1)	8 (13.1)	
Breslow thickness				0.788
≤1.0 mm	6 (3.9)	2 (2.5)	3 (4.9)	
1.1–2.0 mm	0 (0.0)	0 (0.0)	0 (0.0)	
2.1–4.0 mm	5 (3.2)	2 (2.5)	3 (4.9)	
>4.0 mm	15 (9.6)	5 (6.3)	6 (9.8)	
Unknown	128 (83.1)	70 (88.6)	49 (80.3)	
Ulceration				0.639
Yes	38 (24.7)	18 (22.8)	13 (21.3)	
No	58 (37.7)	26 (32.9)	28 (45.9)	
Unknown	58 (37.7)	35 (44.3)	20 (32.8)	
Location metastases				0.007
Locoregional	33 (21.4)	9 (11.4)	19 (31.1)	
Distant	68 (44.2)	42 (53.2)	20 (32.8)	
Both	53 (34.4)	28 (35.4)	22 (36.1)	
Number of metastases				0.614
1 lesion	10 (6.5)	6 (7.6)	3 (4.9)	
2–5 lesions	21 (13.6)	13 (16.4)	6 (9.8)	
6–10 lesions	3 (1.9)	3 (3.8)	0 (0.0)	
>10 lesions	63 (40.9)	35 (44.3)	24 (39.3)	
Unknown	57 (37.0)	22 (27.8)	28 (45.9)	
BRAF-mutation				<0.001
Present	106 (68.8)	36 (45.6)	61 (100.0)	
Absent	45 (29.2)	41 (51.9)	0 (0.0)	
Unknown	3 (1.9)	2 (2.5)	0 (0.0)	
LDH				0.095
≤ULN ^3^	115 (74.7)	62 (78.5)	43 (70.5)	
>ULN (>250 U/L)	34 (22.1)	14 (17.7)	18 (29.5)	
Unknown	55 (35.7)	30 (38.0)	16 (26.2)	
S100				0.328
≤ULN	40 (26.0)	21 (26.6)	18 (29.5)	
>ULN (>0.10 ug/L)	59 (38.3)	28 (35.4)	27 (44.3)	
Unknown	55 (35.7)	30 (38.0)	16 (26.2)	
Sequence systemic therapy				0.006
First Line	107 (69.5)	47 (59.5)	49 (80.3)	
Second line	29 (18.8)	23 (29.1)	5 (8.2)	
Third line	11 (7.1)	4 (5.1)	6 (9.8)	
≥ Fourth line	7 (4.5)	5 (6.3)	1 (1.6)	
Type systemic therapy				
ICI	79 (51.3)			
Targeted therapy	61 (39.6)			
Other/unknown	14 (9.1)			
Best response to systemic therapy				0.027
Stable disease	68 (44.2)	33 (41.8)	27 (44.3)	
Partial response	71 (46.1)	42 (53.2)	25 (41.0)	
Complete response	5 (3.2)	3 (3.8)	1 (1.6)	
Unknown	9 (5.8)	1 (1.3)	8 (13.1)	
Status of disease prior to surgery				0.007
Progressive disease	71 (46.1)	45 (57.0)	20 (32.8)	
Stable disease	45 (29.2)	19 (24.1)	19 (31.1)	
Partial response	29 (18.8)	14 (17.7)	15 (24.6)	
Unknown	9 (5.8)	1 (1.3)	7 (11.5)	
Location surgery				0.685
(Sub)cutaneous/LN	127 (82.5)	56 (77.8)	46 (80.7)	
Visceral	27 (17.5)	16 (22.2)	11 (19.3)	
Complication surgery				0.029
None	122 (79.2)	67 (87.0)	42 (68.9)	
Transient	22 (14.3)	6 (7.8)	15 (24.6)	
Requiring intervention	7 (4.5)	4 (5.2)	3 (4.9)	
Permanent damage	1 (0.6)	0 (0.0)	1 (1.6)	
Death	0 (0.0)	0 (0.0)	0 (0.0)	
Status of disease at first follow-up after surgery				0.459
Progressive disease	26 (16.9)	12 (15.4)	11 (18.0)	
Stable disease	49 (31.8)	28 (35.9)	16 (26.2)	
Partial response	18 (11.7)	18 (11.7)	6 (9.8)	
Complete response	49 (31.8)	22 (28.2)	25 (41.0)	
Unknown	12 (7.8)	4 (5.1)	3 (4.9)	

^1^ Difference between group of patients treated with immune checkpoint inhibitors (ICI) versus patients treated with targeted therapy (TT); ^2^ Melanoma of Unknown Primary (MUP); ^3^ Upper limit of normal (ULN).

**Table 2:** (**a**) Univariate analyses (significant results). (**b**) Multivariate analyses.

**a. Univariate Analyses (Significant Results).**						
	**Overall Survival**			**Progression-Free Survival**		
**Variable**	**HR**	**95% CI**	***p***	**HR**	**95% CI**	***p***
Time between primary tumor and current episode			0.030			0.476
≤1 year	Ref			Ref		
>1 and ≤5 years	0.32	0.15–0.71	0.005	0.65	0.36–1.17	0.150
>5 and ≤10 years	0.50	0.23–1.10	0.083	0.91	0.49–1.68	0.753
>10 years	0.59	0.25–1.39	0.224	0.85	0.43–1.69	0.650
Systemic therapy						
ICI	Ref			Ref		
Targeted therapy	1.65	0.89–3.07	0.115	1.56	0.97–2.49	0.066
Treatment sequence			0.609			0.015
First line	Ref			Ref		
Second line	0.67	0.30–1.51	0.334	0.42	0.21–0.86	0.017
Third line	1.42	0.55–3.64	0.465	1.79	0.88–3.65	0.109
Fourth line or more	0.73	0.10–5.40	0.761	0.53	0.13–2.18	0.380
Duration of systemic treatment						
≤12 months	Ref			Ref		
>12 months	0.60	0.24–1.53	0.288	0.40	0.18–0.86	0.020
Status of disease prior to surgery			0.003			0.004
PR	Ref			Ref		
SD	1.93	0.54–6.96	0.313	1.19	0.55–2.54	0.663
PD	4.82	1.47–15.83	0.009	2.37	1.18–4.75	0.015
Status of disease after surgery			<0.001			<0.001
CR/PR	Ref			Ref		
SD	3.08	1.29–7.38	0.012	2.95	1.66–5.23	<0.001
PD	11.39	4.73–27.47	<0.001	24.20	10.40–56.32	<0.001
Location surgery						
Subcutaneous/LN	Ref			Ref		
Visceral	2.02	1.02–3.94	0.045	1.07	0.60–1.88	0.825
**b. Multivariate analyses.**						
	**Overall Survival**			**Progression-free survival**		
**Variable**	**HR**	**95% CI**	***p***	**HR**	**95% CI**	***p***
Systemic therapy						
ICI	Ref			Ref		
Targeted therapy	3.25	1.48–7.14	0.003	1.89	1.08–3.32	0.026
Status of disease prior to surgery			0.051			0.064
PR	Ref			Ref		
SD	0.69	0.13–3.71	0.669	0.33	0.11–1.00	0.051
PD	2.87	0.55–15.02	0.212	0.69	0.23–2.08	0.514
Status of disease after surgery			<0.001			<0.001
CR/PR	Ref			Ref		
SD	6.05	1.64–22.33	0.007	6.61	2.63–16.59	<0.001
PD	18.62	4.54–76.42	<0.001	37.46	12.25–114.51	<0.001
Duration of systemic treatment						
≤12 months	Ref			Ref		
>12 months	0.34	0.09–1.32	0.119	0.40	0.17–0.97	0.042

## Supplementary Materials

The following are available online at https://www.mdpi.com/2072-6694/12/5/1176/s1. Figure S1: Responses, Figure S2: PFS and OS, Figure S3: PFS and OS per best response to either ICI or targeted therapy (TT), Figure S4: PFS and OS per most recent response prior to surgery in patients treated with either ICI or TT, Figure S5: PFS and OS per status of disease at first follow-up, Figure S6: PFS and OS per location of surgical resection in patients treated with either ICI or TT, Table S1: Univariate analyses.
