# Supplementary material for: Surgery for Unresectable Stage IIIC and IV Melanoma in the Era of New Systemic Therapy
Source: Cancers (Basel). 2020 May 7;12(5):1176. doi: 10.3390/cancers12051176 (PMC7281176; doi:10.3390/cancers12051176)
Supplement: Supplementary file 1 [file cancers-12-01176-s001.pdf]

Supplementary Materials

Surgery for Unresectable Stage IIIC and IV Melanoma in the Era of New Systemic Therapy

Stephanie A. Blankenstein, Maureen J.B. Aarts, Franchette W.P.J. van den Berkmortel, Marye J. Boers-Sonderen, Alfons J.M. van den Eertwegh, Margreet G. Franken, Jan Willem B. de Groot, John B.A.G. Haanen, Geke A.P. Hospers, Ellen Kapiteijn, Djura Piersma, Rozemarijn S. van Rijn, Karijn P.M. Suijkerbuijk, Albert J. ten Tije, Astrid A.M. van der Veldt, Gerard Vreugdenhil, Michel W.J.M. Wouters and Alexander C.J. van Akkooi

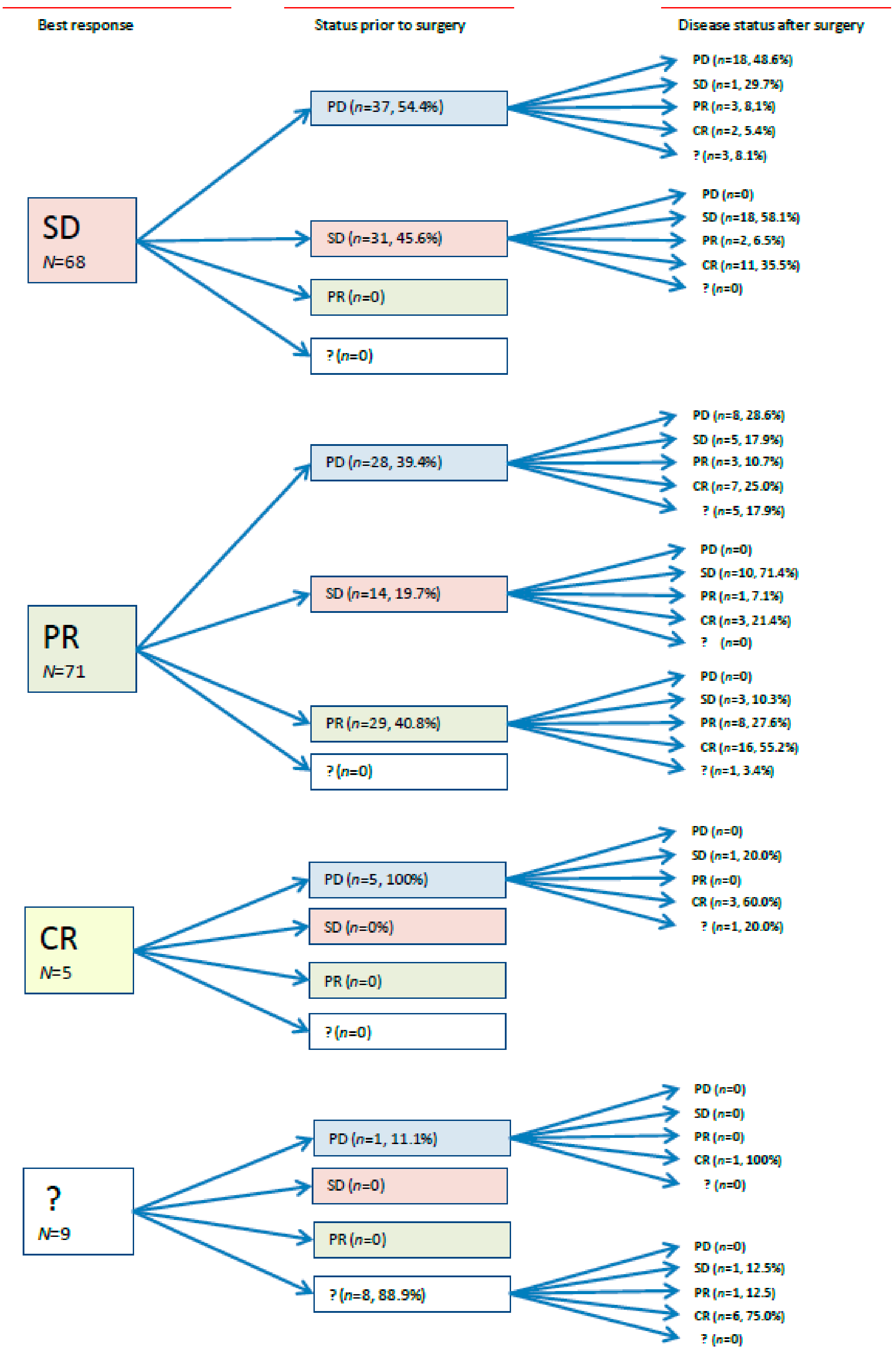

Figure S1. Responses.

Table S1. (**a**) Response per type of ICI treatment. (**b**) Response per type of TT treatment.

a. Response Per Type of ICI Treatment.

Characteristic	PD-1 (*n* = 38) *n* (%)	CTLA-4 (*n* = 35) *n* (%)	Both (*n* = 6) *n* (%)
Best response to systemic therapy			
Stable disease	11 (28.9%)	19 (54.3%)	3 (50.0%)
Partial response	26 (68.4%)	13 (37.1%)	3 (50.0%)
Complete response	1 (2.6%)	2 (5.7%)	0 (0.0%)
Unknown	0 (0.0%)	1 (2.9%)	0 (0.0%)
Most recent response to systemic therapy			
Progressive disease	17 (44.7%)	26 (74.3%)	2 (33.3%)
Stable disease	12 (31.6%)	6 (17.1%)	1 (16.7%)
Partial response	9 (23.7%)	2 (5.7%)	3 (50.0%)
Unknown	0 (0.0%)	1 (2.9%)	0 (0.0%)
Status after surgery			
Progressive disease	6 (16.2%)	5 (14.3%)	1 (16.7%)
Stable disease	12 (32.4%)	16 (45.7%)	0 (0.0%)
Partial response	7 (18.9%)	4 (11.4%)	1 (16.7%)
Complete response	9 (24.3%)	9 (25.7%)	4 (66.7%)
Unknown	3 (8.1%)	1 (2.9%)	0 (0.0%)

b. Response Per Type of TT Treatment.

Characteristic	BRAFi (*n* = 30) *n* (%)	BRAFi+MEKi (*n* = 31) *n* (%)
Best response to systemic therapy		
Stable disease	19 (63.3%)	8 (25.8%)
Partial response	10 (33.3%)	15 (48.4%)
Complete response	0 (0.0%)	1 (3.2%)
Unknown	1 (3.3%)	7 (22.6%)
Most recent response to systemic therapy		
Progressive disease	10 (33.3%)	10 (32.3%)
Stable disease	13 (43.3%)	6 (19.4%)
Partial response	6 (20.0%)	9 (29.0%)
Unknown	1 (3.3%)	6 (19.4%)
Status after surgery		
Progressive disease	7 (23.3%)	4 (12.9%)
Stable disease	10 (33.3%)	6 (19.4%)
Partial response	2 (6.7%)	4 (12.9%)
Complete response	9 (30.0%)	16 (51.6%)
Unknown	2 (6.7%)	1 (3.2%)

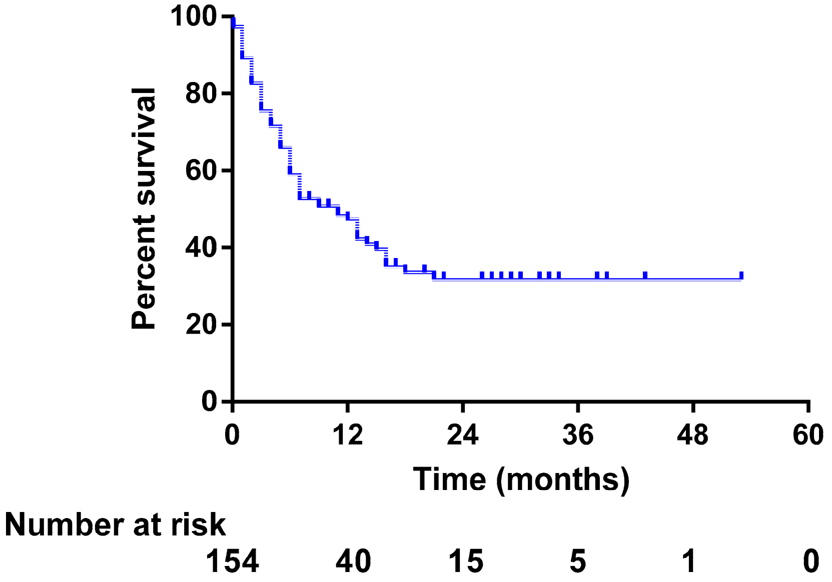

(a)

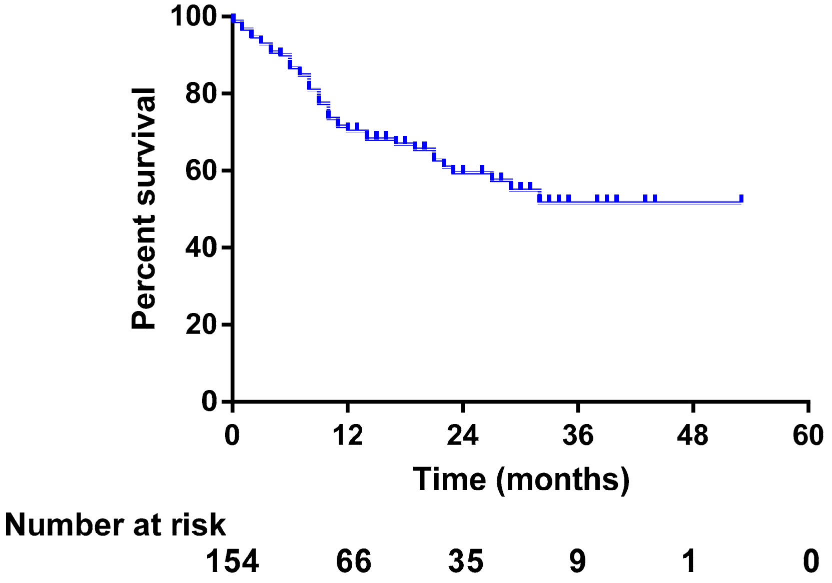

(b)

Figure S2. (**a**) Progression free survival. (**b**). Overall survival.

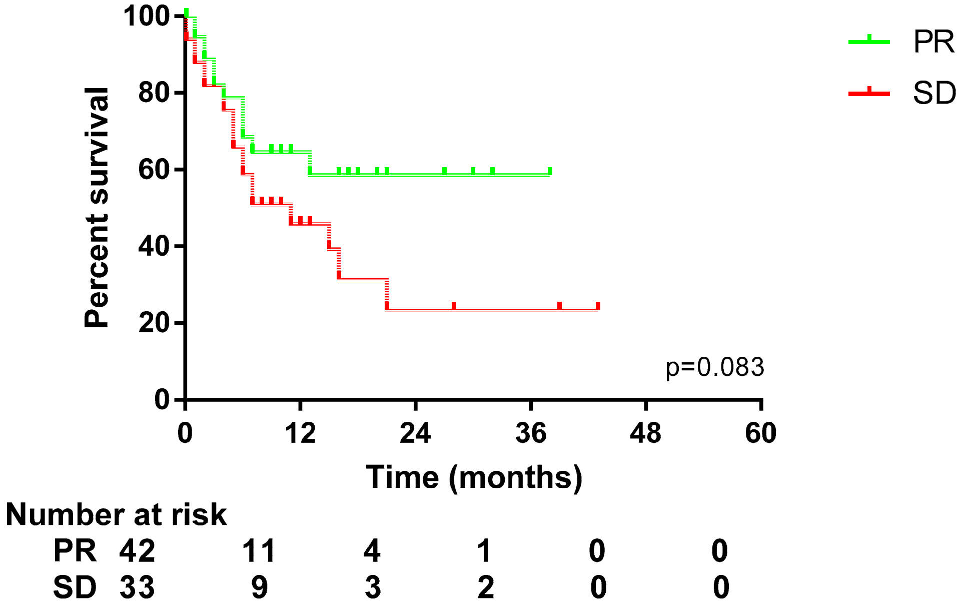
100
80
60
40
20
0
Percent survival
0
12
24
36
48
60
Time (months)
p=0.083
PR
SD
Number at risk
PR 42 11 4 1 0 0
SD 33 9 3 2 0 0

(a)

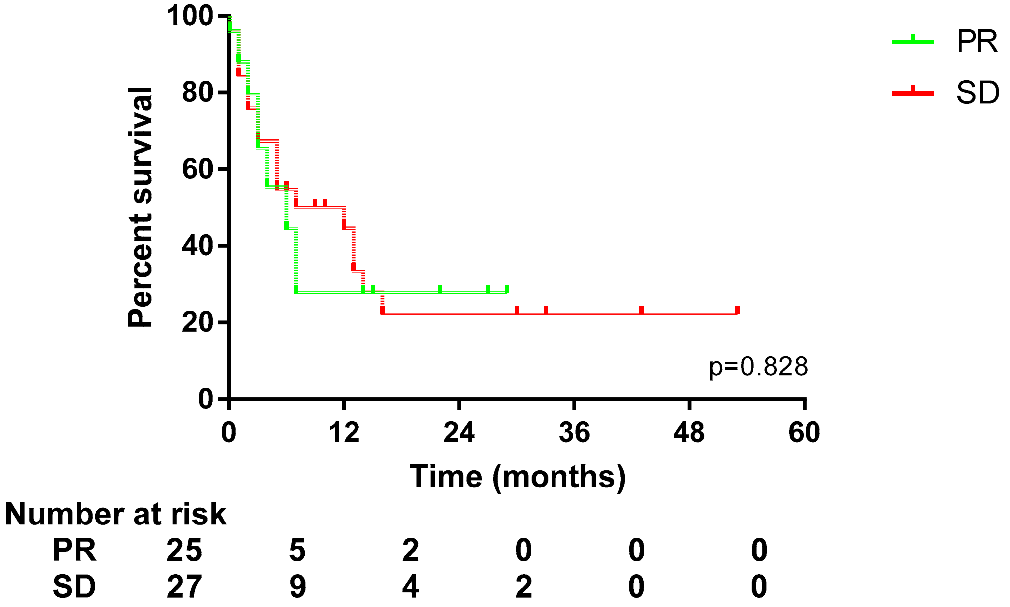
100
80
60
40
20
0
Percent survival
0
12
24
36
48
60
Time (months)
p=0.828
PR
SD
Number at risk
PR 25 5 2 0 0 0
SD 27 9 4 2 0 0

(b)

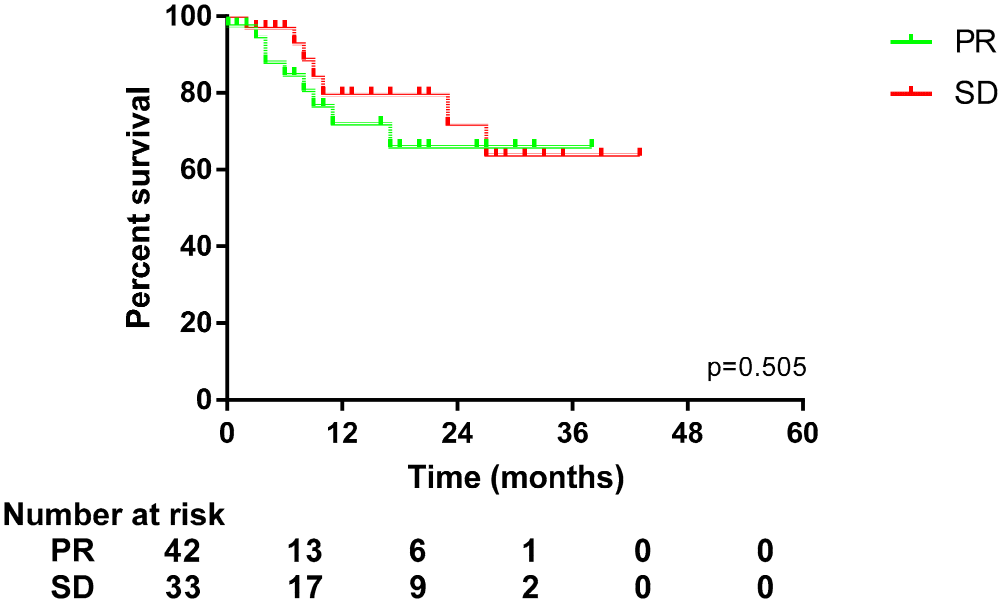

(c)

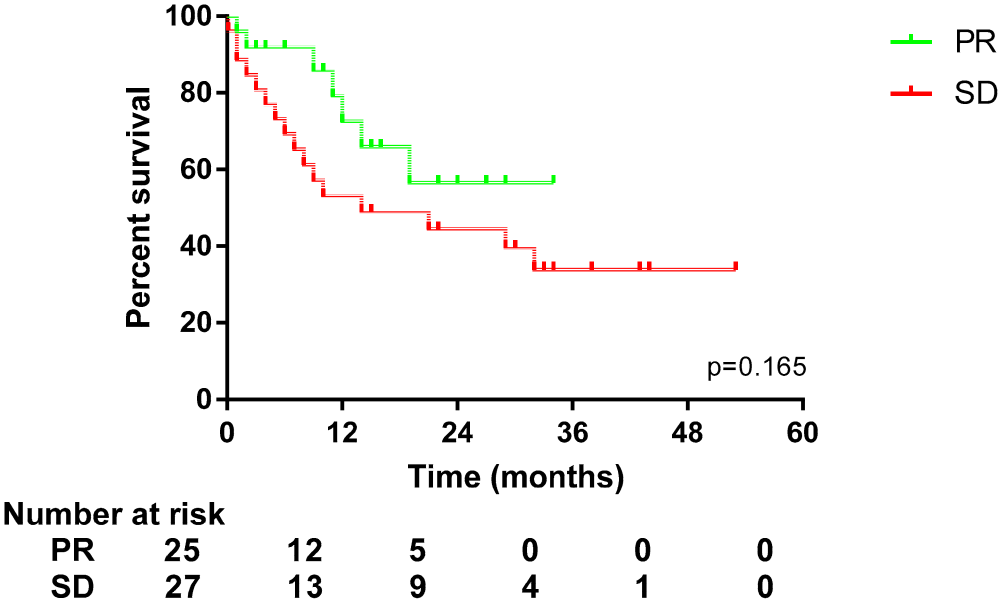

(d)

Figure S3. (**a**) PFS per best response to systemic therapy in patients treated with ICI. (**b**) PFS per best response to systemic therapy in patients treated with TT. (**c**) OS per best response to systemic therapy in patients treated with ICI. (**d**) OS per best response to systemic therapy in patients treated with TT.

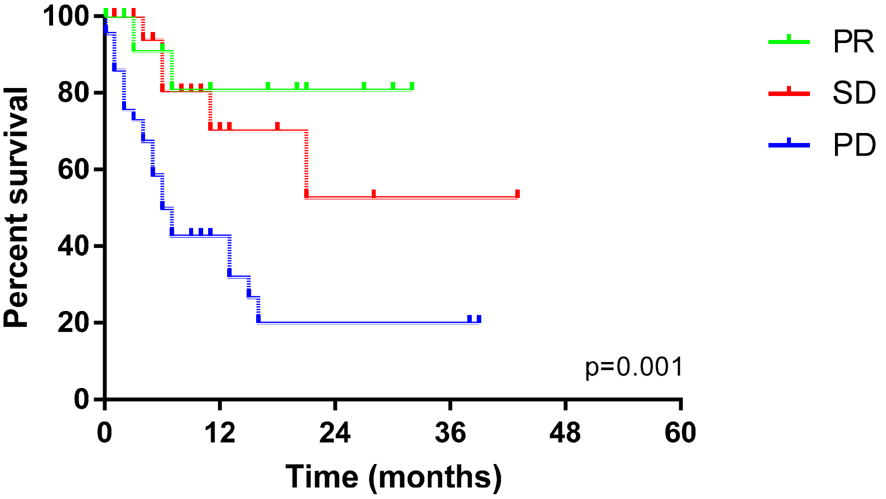

Number at risk

PR	14	6	3	0	0	0
SD	19	7	2	1	0	0
PD	45	8	2	2	0	0

(a)

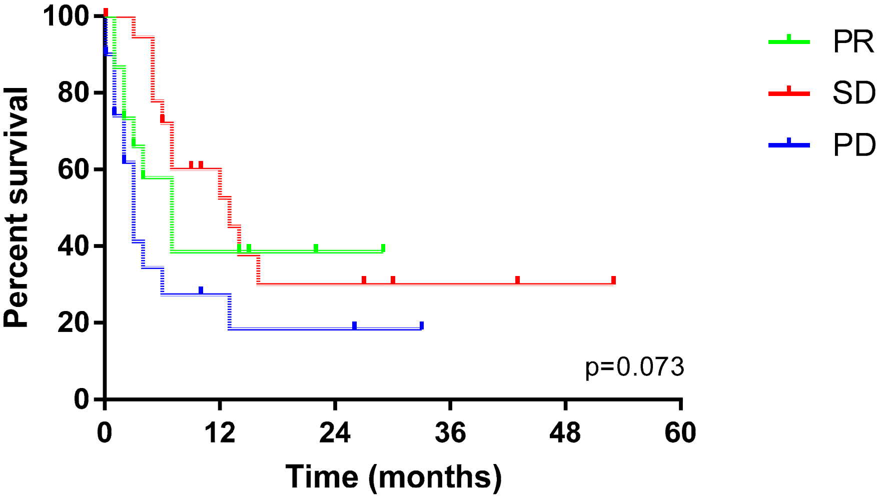

Number at risk

PR	15	4	1	0	0	0
SD	19	8	4	2	0	0
PD	20	3	2	0	0	0

(b)

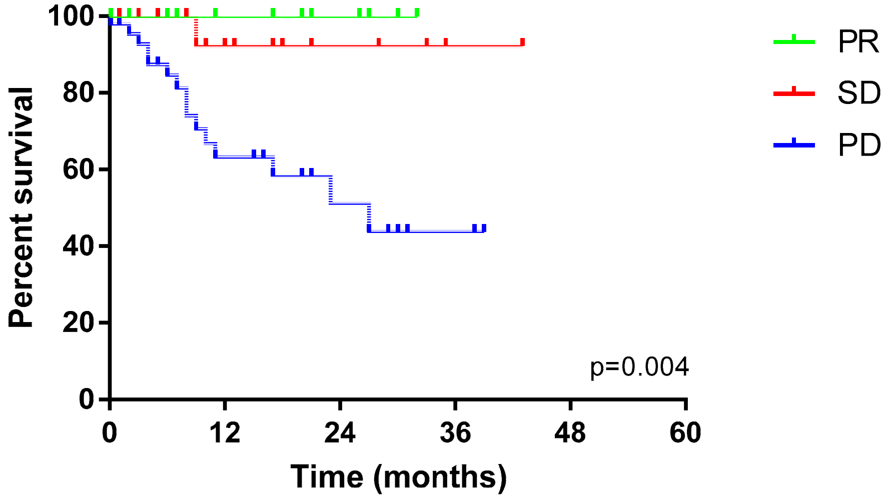

Number at risk

PR	14	7	4	0	0	0
SD	19	9	4	1	0	0
PD	45	15	7	2	0	0

(c)

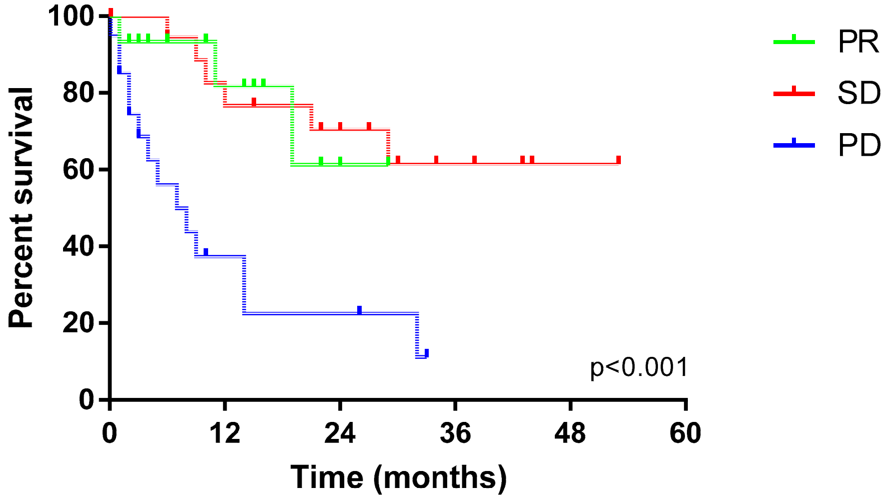

Number at risk

PR	15	7	2	0	0	0
SD	19	13	10	4	1	0
PD	20	5	3	0	0	0

(d)

Figure S4. (**a**) PFS per most recent response prior to surgery in patients treated with ICI. (**b**) PFS per most recent response prior to surgery in patients treated with TT. (**c**) OS per most recent response prior to surgery in patients treated with ICI. (**d**) OS per most recent response prior to surgery in patients treated with TT.

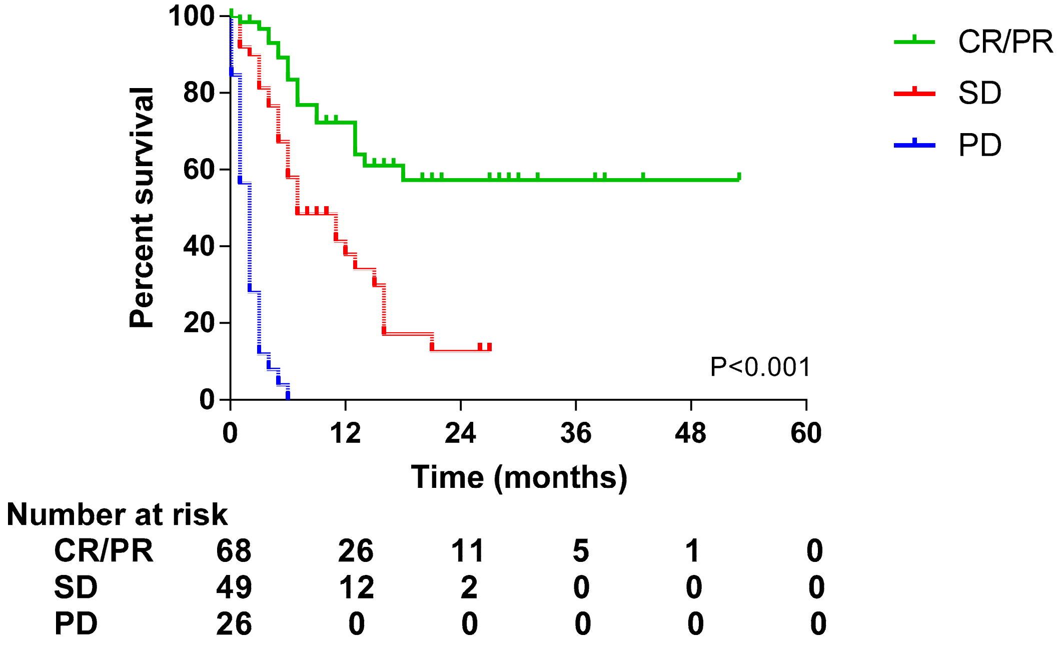

(a)

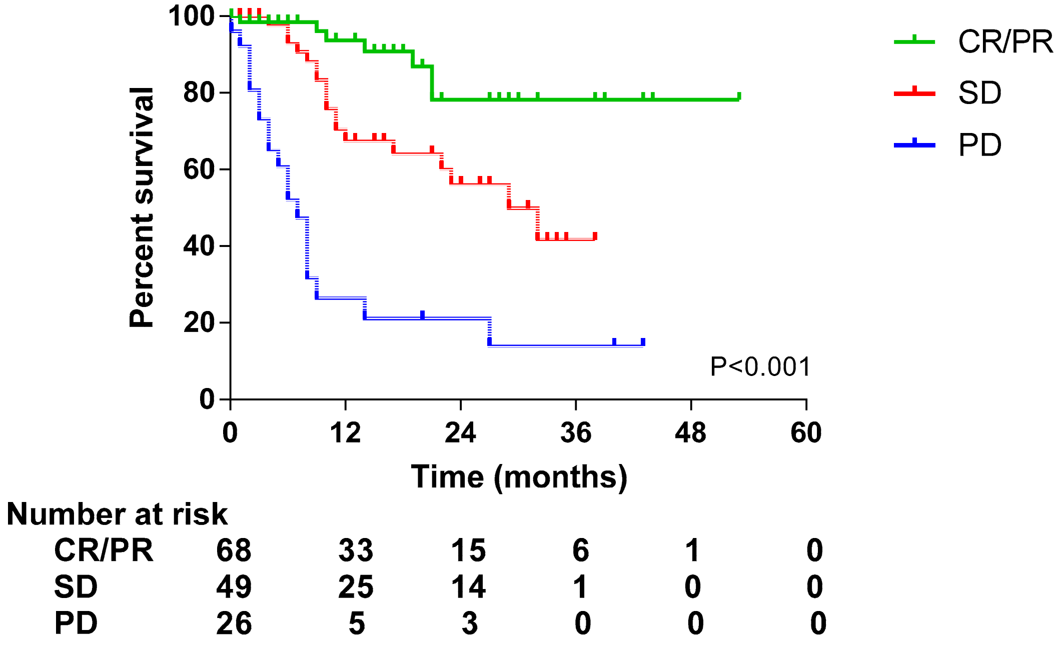

(b)

Figure S5. (**a**) PFS per status of disease at first follow-up after surgery. (**b**) OS per status of disease at first follow-up after surgery.

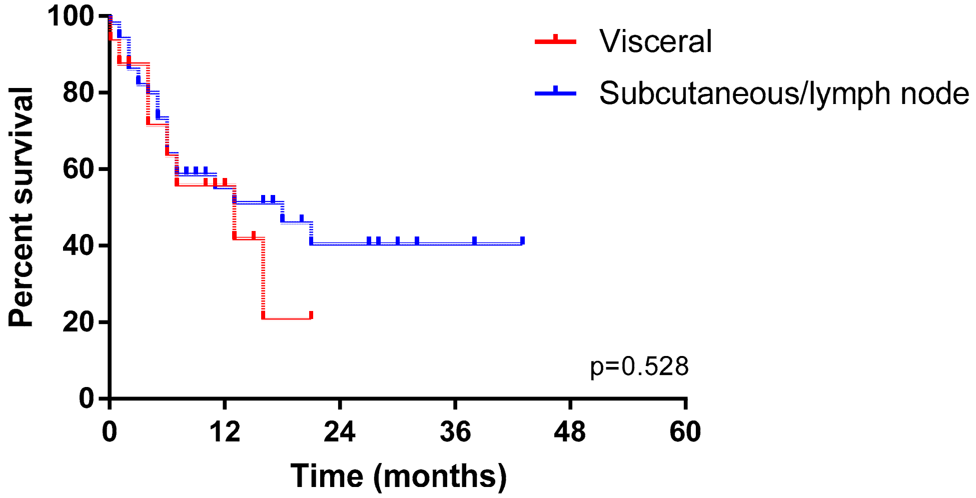

Number at risk

**Visceral**	**16**	**5**	**0**	**0**	**0**	**0**
**SC/LN**	**56**	**14**	**6**	**2**	**0**	**0**

(a)

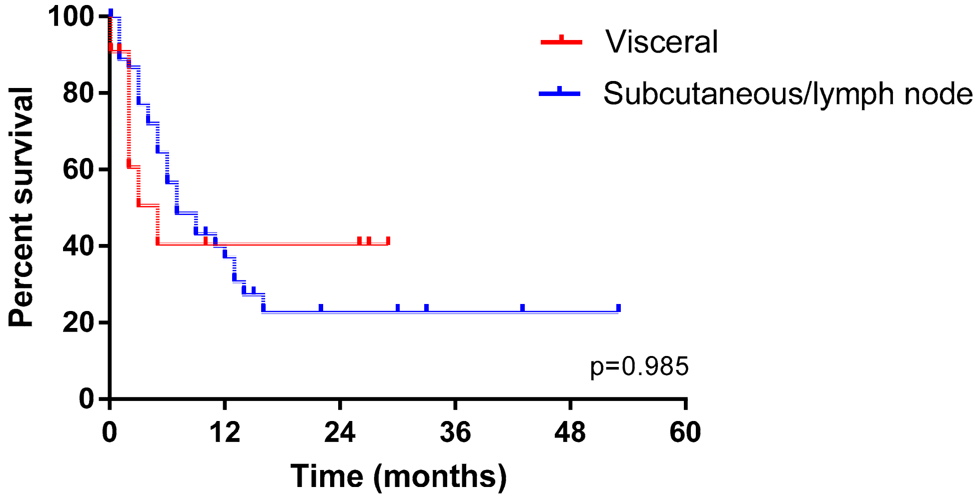

Number at risk

**Visceral**	**11**	**3**	**3**	**0**	**0**	**0**
**SC/LN**	**46**	**13**	**4**	**2**	**1**	**0**

(b)

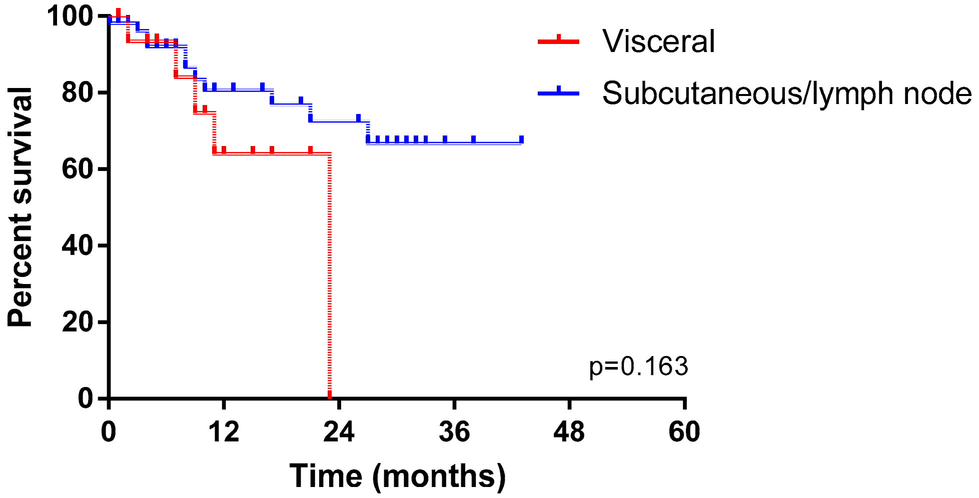

Number at risk

	0	12	24	36	48	60
Visceral	16	5	0	0	0	0
SC/LN	56	24	14	2	0	0

(c)

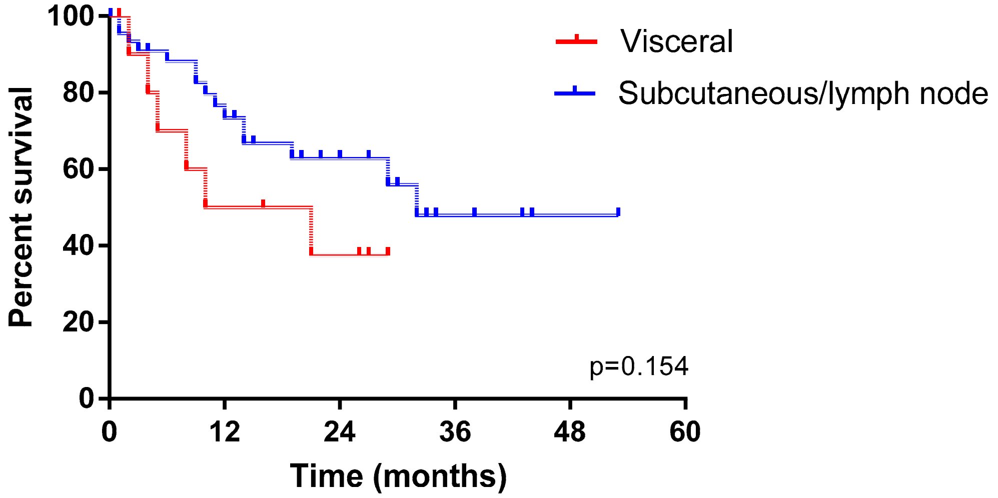

Number at risk

	0	12	24	36	48	60
Visceral	11	5	3	0	0	0
SC/LN	46	24	13	4	1	0

(d)

Figure S6. (a) PFS per location of surgical resection in patients treated with ICI. Supp. (b) PFS per location of surgical resection in patients treated with TT. Supp. (c) OS per location of surgical resection in patients treated with ICI. (d) OS per location of surgical resection in patients treated with TT.

Table S2. Univariate analyses.

	Overall Survival			Progression-Free Survival		
**Variable**	**HR**	**95% CI**	***p***	**HR**	**95% CI**	***p***
Sex						
Female	Ref			Ref		
Male	0.84	0.47–1.50	0.555	1.17	0.74–1.83	0.502
Age at start current episode	1.00	0.97–1.021	0.785	0.99	0.97–1.01	0.274
WHO performance status			**0.021**			0.514
0	Ref			Ref		
1	1.44	0.74–2.81	0.281	1.11	0.66–1.85	0.699
2 (*n* = 4)	5.93	1.29–27.33	**0.023**	2.54	0.61–10.68	0.203
3 (*n* = 1)	9.04	1.14–71.42	**0.037**	2.95	0.40–21.79	0.289
Location primary tumor			0.679			0.679
Extremity	Ref			Ref		
Acral	0.88	0.26–2.97	0.834	0.42	0.13–1.36	0.147
Trunk	0.77	0.38–1.55	0.458	0.95	0.57–1.61	0.860
Head/Neck	0.52	0.12–2.23	0.377	1.21	0.54–2.76	0.643
Unknown primary	1.27	0.57–2.81	0.564	0.52	0.24–1.13	0.097
Type			0.798			**0.033**
Superficial spreading	Ref			Ref		
Nodular	1.46	0.61–3.48	0.394	1.94	1.06–3.56	**0.033**
Acral lentiginous	1.04	0.24–4.57	0.963	0.38	0.09–1.58	0.181
Ulceration						
No	Ref			Ref		
Yes	0.94	0.41–2.12	0.875	0.72	0.39–1.33	0.295
Time between primary tumor and current episode			**0.030**			0.476
≤1 year	Ref			Ref		
>1 and ≤5 years	0.32	0.15–0.71	**0.005**	0.65	0.36–1.17	0.150
>5 and ≤10 years	0.50	0.23–1.10	0.083	0.91	0.49–1.68	0.753
>10 years	0.59	0.25–1.39	0.224	0.85	0.43–1.69	0.650
Site metastases			0.471			0.495
Locoregional	Ref			Ref		
Distant	1.53	0.66–3.57	0.325	0.77	0.42–1.40	0.383

Both	1.11	0.45–2.70	0.827		1.03	0.57–1.86	0.922
BRAF mutation							
Negative	Ref				Ref		
Positive	1.35	0.67–2.73	0.402		1.03	0.62–1.71	0.901
S100							
Normal	Ref				Ref		
Elevated (>0.10ug/L)	1.05	0.49–2.23	0.902		0.92	0.52–1.62	0.769
LDH							
Normal	Ref				Ref		
Elevated (>250 U/L)	1.32	0.67–2.60	0.430		0.92	0.52–1.62	0.769
Treatment sequence			0.609				**0.015**
First line	Ref				Ref		
Second line	0.67	0.30–1.51	0.334		0.42	0.21–0.86	**0.017**
Third line	1.42	0.55–3.64	0.465		1.79	0.88–3.65	0.109
Fourth line or more	0.73	0.10-5.40	0.761		0.53	0.13–2.18	0.380
Systemic therapy							
ICI	Ref				Ref		
Targeted therapy	1.65	0.89–3.07	0.115		1.56	0.97–2.49	0.066
Duration of systemic treatment							
≤12 months	Ref				Ref		
>12 months	0.60	0.24–1.53	0.288		0.40	0.18–0.86	**0.020**
Best response to systemic therapy							
CR/PR	Ref				Ref		
SD	1.50	0.81–2.78	0.202		1.56	0.91–2.34	0.121
Status of disease prior to surgery			**0.003**				**0.004**
PR	Ref				Ref		
SD	1.93	0.54–6.96	0.313		1.19	0.55–2.54	0.663
PD	4.82	1.47–15.83	**0.009**		2.37	1.18–4.75	**0.015**
Status of disease after surgery			**<0.001**				**<0.001**
CR/PR	Ref				Ref		
SD	3.08	1.29–7.38	**0.012**		2.95	1.66–5.23	**<0.001**
PD	11.39	4.73–27.47	**<0.001**		24.20	10.40–56.32	**<0.001**
Location surgery							
Subcutaneous/LN	Ref				Ref		

Visceral	2.02	1.02–3.94	**0.045**	1.07	0.60–1.88	0.825

© 2020 by the authors. Licensee MDPI, Basel, Switzerland. This article is an open access article distributed under the terms and conditions of the Creative Commons Attribution (CC BY) license (http://creativecommons.org/licenses/by/4.0/).
